# Public Opinion Manipulation on Social Media: Social Network Analysis of Twitter Bots during the COVID-19 Pandemic

**DOI:** 10.3390/ijerph192416376

**Published:** 2022-12-07

**Authors:** Zixuan Weng, Aijun Lin

**Affiliations:** School of Journalism and Communication, Jinan University, Guangzhou 510632, China

**Keywords:** social bot, public opinion manipulation, social network, COVID-19, Latent Dirichlet Allocation, natural language processing, human-machine communication, Botometer, Wuhan lab, China

## Abstract

Social media is not only an essential platform for the dissemination of public health-related information, but also an important channel for people to communicate during the COVID-19 pandemic. However, social bots can interfere with the social media topics that humans follow. We analyzed and visualized Twitter data during the prevalence of the Wuhan lab leak theory and discovered that 29% of the accounts participating in the discussion were social bots. We found evidence that social bots play an essential mediating role in communication networks. Although human accounts have a more direct influence on the information diffusion network, social bots have a more indirect influence. Unverified social bot accounts retweet more, and through multiple levels of diffusion, humans are vulnerable to messages manipulated by bots, driving the spread of unverified messages across social media. These findings show that limiting the use of social bots might be an effective method to minimize the spread of conspiracy theories and hate speech online.

## 1. Introduction

Social bots have influenced public opinion about COVID-19 in social media. During the COVID-19 pandemic, the responsible and appropriate use of social media has become a fast and effective way to disseminate critical information during the COVID-19 pandemic [1]. Meanwhile, the vast spread of misinformation, conspiracy theories and fake news on social media is also a pressing question. Academics have used the infodemic to describe their research on spreading COVID-19-related information, online search behavior, or spreading misinformation on Twitter [2,3,4,5,6,7]. The spread of the infodemic not only fuels the rise of racist attitudes and behaviors, but also poses a significant global risk by putting both the health of the population and the ability of governments to implement effective preventive measures at risk. Moreover, as computer algorithms, social bots can generate content and interact with humans on social media to mimic and possibly manipulate humans and their behavior [8,9]. In the context of the widespread influence of social media on individual opinions and behaviors, social bots could be used to massively spread misinformation, fake news, hate speech, and manipulate public opinion [10,11,12,13]. We need to reconsider the nature and function of social bots in light of the COVID-19 outbreak.

Based on the above background, this study selected the tweets during the Wuhan lab-leak theory as analysis samples to conduct social network analysis to find out and visualize the role of social bots and the path of human-bot communication.

## 2. Literature Review

### 2.1. COVID-19 and Social Networks

During the COVID-19 pandemic, information in social networks can influence people’s perceptions, attitudes, and behaviors regarding health issues. The rising popularity of social networks and media increased rapidly when people were forced to be isolated following social distancing norms [14,15]. The COVID-19 pandemic has caused extensive disturbance in individuals’ beliefs and societal structures. People turn online for alternative cognitive and social structures when faced with these distractions [16]. However, governments and public health agencies were slow to disseminate information online and build public trust, leaving room for the spread of COVID-19 misinformation and conspiracy theories [17]. Social media platforms, such as YouTube and Twitter, providing direct access to unprecedented content, can amplify rumors and suspicious news [18]. For example, many fictional stories were popular on Twitter: the 5G network activates the virus; the pandemic is a hoax created by a global conspiracy; the virus is a biological weapon intentionally released by the Chinese; or Bill Gates is using it as cover to activate a global surveillance system [19]. A popular view linked 5G to the spread of COVID-19, leading to the burning of 5G towers in the UK [20]. Moreover, the spread of stigmatized negative emotional information on social media can create cyber racism. COVID-19 is driving the rise of online Sinophobia, and the Web is being exploited for spreading conspiracy theories targeting Chinese and Asians, as COVID-19 is believed to have originated in China [21]. Studies have analyzed sentiment on Twitter associated with terms such as “Chinese virus”, “Wuhan virus”, and “Chinese coronavirus”, and have shown that the majority of tweets analyzed were negative and tinged with feelings of fear, sadness, anger, and disgust. The use of slander and profane words was high [22].

Research proved that fake news tends to spread faster than verified news [23]. The global epidemic of misinformation on social media platforms poses serious problems for public health [7]. More importantly, health-related misinformation threatens public compliance with public health safeguards. Studies have shown that belief in conspiracy theories increases the likelihood that individuals will ignore public health interventions and safeguards from the government or public health agencies [24,25], for example, less social distancing and hand washing [26]. Thus, monitoring the spread of misinformation on social media is critical to understand the evolution of opinions that may negatively impact public health [27].

### 2.2. Social Bots and Humans on Social Media

Social bots have made an impact in multiple areas of human concern. Research has identified interference with social bots in the UK’s Brexit vote [28] and the US [29], French [30], and German elections [9]. Studies have also reported the impact of social bots on topics such as cryptocurrencies [31], stock markets [32], and climate change [33], and actively engaged in public health discussions about vaccination [34], the COVID-19 pandemic [12,35], and cannabis [36]. Social bots have also been involved in COVID-19 vaccine-related discussions [37]. In contrast to bots in anti-vaccination networks, bots in pro-vaccination networks influence the dissemination of pro-vaccination messages [38].

Social bots also played a role in distorting public opinion and emotional expression. They have the potential to actively amplify negative human emotions, such as in the US discussion about COVID-19, where social bots managed to trigger a bot-to-human spread of anger [39]. Previous studies examined the role of social bots in the spread of the COVID-19 infodemic and the diffusion of non-credible information such as “5G” and “Bill Gates” conspiracy theories and content related to “Trump” and the “WHO” by analyzing retweet networks and retweet items [40,41]. Bots increased the exposure of negative and inflammatory content in online social systems [42]. Recent research suggests bots are more in tune with political content than health-related content. While humans focus more on cover-up and social distancing utterances, bots engage in more political topics [43].

### 2.3. Social Media Data and Social Bots Detection

Social media big data analytics is an effective large-scale tool that can identify public health issues and improve national public health policies—for example, COVID-19 vaccination [44]. Researchers also used social media data on Twitter to assess children’s exposure to violence during the COVID-19 pandemic [45]. However, misinformation mixed in with social media data is a serious problem for both ordinary users and researchers. Misinformation based on the Internet and social media remain a common and significant problem during the pandemic. The findings suggest unverified accounts contain more erroneous medical information than verified ones [46]. Therefore, it is still necessary for us to consider the influence of unverified bot accounts and proceed through bot detection tools.

Bot detection is crucial in a world where social media is increasingly vital as our communication channel. Techniques used to develop bot detection models use features such as metadata of tweets or digital fingerprinting of accounts [47]. Unsupervised methods are more robust than supervised methods because they do not depend on the ground truth quality. The research proposed an unsupervised approach that uses features extracted from the retweet patterns of accounts and a clustering algorithm to distinguish bots from humans [48]. Bot detection is by no means an exact science. Within the scope of this study, we cannot assess which tool is “better” at making correct predictions about the actual amount of automation [49]. The tool employed in this study, Botometer, is an exceptionally well-developed bot detection tool used by many influential studies and organizations, such as the PEW Research Center.

Due to the uncertainty of the post-pandemic era and the possibility of future pandemics, it is essential to understand the drivers of misinformation and conspiracy theories and explore strategies to mitigate them, especially if there may be technological algorithmic elements, such as social bots. First, while some studies have highlighted the theoretical mechanisms by which social media influences COVID-19 conspiracy theories [16], the central issue remains empirical research. Recent empirical study has conducted social network analysis on Twitter data for COVID-19 and 5G conspiracy theories but is limited by not identifying social bot accounts [21], thus ignoring essential drivers of conspiracy theories.

Second, in the early stages of the COVID-19 pandemic, researchers’ topic and sentiment analysis of tweets can help understand the public’s sentiments, beliefs, and thoughts [50]. However, the potential for future pandemics to continue to pose secondary problems, such as the massive spread of rumors, conspiracy theories, and disinformation on social media, forces us to look at the multiple factors that may be driving these problems. Third, existing research lacks the identification of information flow and the identification of network nodes of influential people that stimulate virality. Finally, the visual presentation and social network analysis of human-bot social networks during the COVID-19 pandemic also need to be strengthened.

In this article, we aim to explore and visualize the communication patterns between social bots and humans on Twitter during the prevalence of the Wuhan lab-leak theory. More specifically, we will answer the following research questions: First, what are the structural characteristics of social bots and human accounts in social networks? Second, what role do social bots play in the Wuhan lab-related topics? Finally, how do tweets (including conspiracy theories and hate speech) spread between humans and social bots?

**Figure 1:**
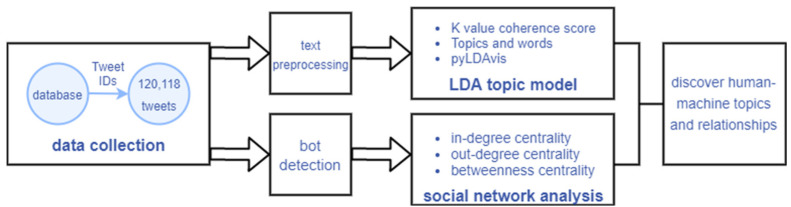
Steps used in this study.

A diagram of the analysis process is shown in Figure 1. First, using the Twitter IDs gathered in the database, we synthesized 120,118 tweets. Second, we performed text preprocessing on contents and social bot detection on the accounts. We analyzed the content using the LDA topic model at the content level. At the relational level, we use social network analysis to calculate the network structure features of all accounts. Finally, we expect the abovementioned analysis process to help us identify the unique human-machine themes and interactions in the COVID-19 pandemic discussions.

## 3. Materials and Methods

### 3.1. Data

In this study, Twitter was selected as the data source, which provides an open application programming interface (API) to provide researchers with a large amount of publicly available information upon specific request. As this study deals with historical data during the COVID-19 pandemic, a Twitter dataset collected by the Information Science Institute at the University of Southern California was used. The dataset contains Tweet ID related to specific keywords and accounts through Twitter’s streaming API and Tweepy, which has stored Covid-related tweet ids since 28 January 2020, with nearly 2 billion tweets stored so far and continuously updated [51]. This study selected the tweet data from 23 May 2021, to 29 May 2021.

**Figure 2:**
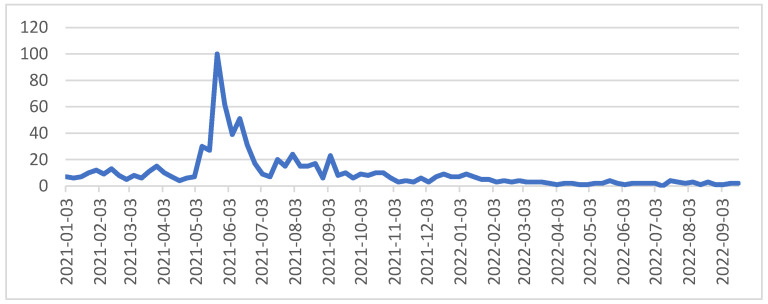
“Wuhan Lab” keywords Google trends.

Regarding Google Trends, “Wuhan lab” is more prominent from 23 May 2021, to 29 May 2021, displayed in Figure 2. Among the issues related to China, the word “Wuhan lab” has become the object of “stigmatization” against China in the international infodemic.

This study extracted the data of Tweet IDs in the database from 23 May 2021, to 29 May 2021, and adopted Python programming to set random seeds for random sampling of the Tweet IDs within every hour according to the proportion of 1%, and then synthesized the data through Twarc. Twarc is a command-line tool for collecting Twitter data in JSON format from Twitter API [52,53]. After filtering, we end up with 120,118 tweets.

### 3.2. Social Bot Detection

The Twitter account’s bot score is calculated using the Botometer service, which assesses the extent to which the account exhibits characteristics similar to social bots [54]. Botometer examines six main features: personal data, friends, social networks, temporal activity patterns, language, and emotions. Through machine learning of more than 1000 features of the information communication network, such as various descriptors, user metadata, friend statistics, temporal patterns of activities, parts of speech and sentiment analysis, the bot score between 0 and 1 is obtained. Higher scores indicate a higher likelihood of automation. We excluded unauthorized users and undetected users in this study.

In the experimental environment, Botometer works really well. V4 has an AUC (area under the receiver operating characteristic curve) of 0.99, suggesting that the model can distinguish bot and human accounts with very high accuracy [55]. Bot score dichotomy is adopted in this study, and accounts with scores above a threshold are considered social bots. In the literature, 0.5 is the most common choice [10]. The Botometer, however, is not flawless and may misclassify accounts owing to a variety of factors. There is a certain percentage of false negative and false positive accounts [56], depending on the data set on which the training is performed [55]. It has been pointed out that Botometer scores are imprecise when estimating bots, especially in different languages. Based on this study’s considerable data, we take 0.5 as the threshold for determining social bot accounts. At the same time, due to the instantaneous bot scores, we carried out social bot detection as soon as possible after obtaining the tweet data to ensure the accuracy of the data.

### 3.3. LDA Topic Model

Firstly, the text preprocessing of the data is carried out, and the complete text data of each English-language tweet is extracted by Python programming. The data needed to establish the topic model has been tokenized, and removed the stop words, the URL, and the non-alphabetic characters. Secondly, the probability topic modelling is carried out through Latent Dirichlet Allocation (LDA) [57]. Finally, to ensure the effectiveness of the topic model, it is necessary to evaluate the calculation results of the topic model. In this study, the coherence score was used to calculate the similarity of words in topics. We visualized the topic modelling results with pyLDAvis to determine the appropriate number of topics.

The LDA topic model is used for extended document analysis to extract and summarize topics from documents. It contains three layers of structure: word, topic, and document. LDA has a good effect in analyzing text semantics and can effectively analyze large-scale unstructured document sets. It is progressively being used for short messages, such as tweets [58]. The topics implied by the LDA topic model are unknown and need to be self-summarized. There has been an explosion of research using LDA topic models in social media [59,60]. LDA can identify topics in YouTube transcripts [61], or carry out a topic-clustering analysis of users’ online comments [62].

### 3.4. Social Network Analysis

The theory of social network analysis holds that society is composed of different actors and forms a network through different connections. A social network comprises a limited set of groups of actors and their relationships [63]. Social network analysis is a research method that uses technical means and tools to analyze the network structure and further reveal social relations. Usually, the relationship data needed to construct social communication network mainly includes “retweet”, “follow”, “like”, and “comment”. This study focuses on the “retweet” relationship. On the one hand, user behaviors such as “following” or “like” are not necessarily closely related to topic diffusion behavior. On the other hand, the “retweet” relationship can more clearly reflect the specific communication path of users on social platforms. This study uses Python client library to clean and process the Twitter data, which is imported into Gephi0.9.2 for visualization.

The specific measurement indicators of the network structure mainly include:

(1) Network scale, that is, the number of nodes and edges contained in the network structure.
(2) Degree centrality. Usually, nodes with a higher degree centrality are in the core position and have a greater right to speak. If the network is directed, two distinct metrics of degree centrality, indegree and outdegree, are specified. The degree in these circumstances is equal to the sum of the indegree and outdegree.
(3) Betweenness centrality. The node with higher betweenness centrality plays a more vital role as a bridge.

In this study, by extracting the retweet relationship between users, the original user is taken as the source node, the retweet relationship is taken as the edge, and the retweeting user is taken as the target node to form a directed network. Gephi’s directed social network analysis graph can obtain the center, node size, and connection relationship of a social network. The human-bot communication network and node connections may then be evaluated.

## 4. Results

### 4.1. Corpus Analysis and Text Preprocessing

Through a self-written Python program, this study removed meaningless words from the top-ranked high-frequency words, such as Corona, virus, coronavirus, Covid, pandemic, still, time, today, and think. Finally, we obtained a set of top 20 high-frequency keywords in tweets. According to Table 1, people have heightened concerned about “vaccines”, “India”, “Wuhan”, “First”, and “China”.

Text preprocessing is carried out through the powerful natural language processing python library NLTK, which comes with various corpus, processing methods, and interfaces. In this study, the NLTK is used to segment the English text of Twitter, remove special symbols such as emoticons, remove stop words, remove hyperlinks, restore word form, and unify the text into lowercase. Text comparison before and after the text preprocessing is shown in Table 2.

### 4.2. LDA Model Analysis

**Figure 3:**
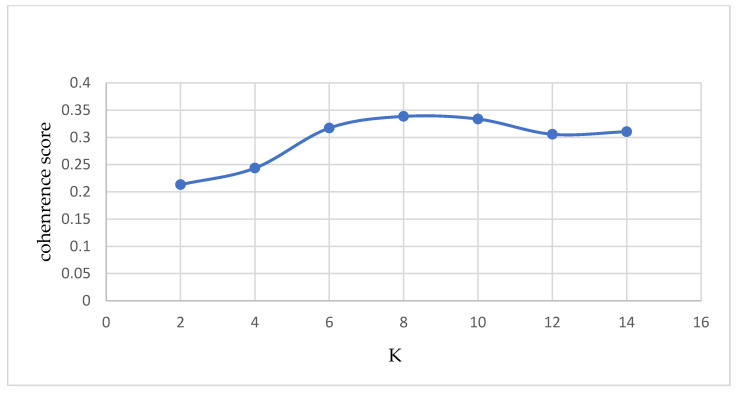
K value coherence score line chart.

According to the principle of the LDA model, the number of topics K of the LDA model needs to be specified by the users themselves, and there is no optimal solution. Hence, users need to judge based on the effectiveness of the model. The coherence score and topic model visualization were used as the criteria to test the model’s validity. A higher coherence score indicates better interpretability and more meaningful topics, which are semantically consistent. Figure 3 shows that the K value should be 8.

As shown in Table 3, the focus of concern for the outbreak-related discussion is the prevention and cure of virus source, the vaccine, social influence, family influence, virus, prevention measures, health effects, the public interests, and other topics of Twitter users.

In previous studies, pyLDAvis was employed to evaluate produced models and visualize the inter-topic distance maps [64,65,66]. The dynamic interactive LDA topic visualization map generated by pyLDAvis can analyze the correlation between research topics. The model is better if the circle in the visualization result is significant and scattered. PyLDAvis can adjust the parameters lambda to control the topic relevance—word correlation. According to the test results of previous studies, the “optimal” value of λ is about 0.6, and its probability of correctly identifying the topics is estimated to be 70% [67]. Therefore, in this study, the value of λ is set as 0.6, and the number of topics is eight.

**Figure 4:**
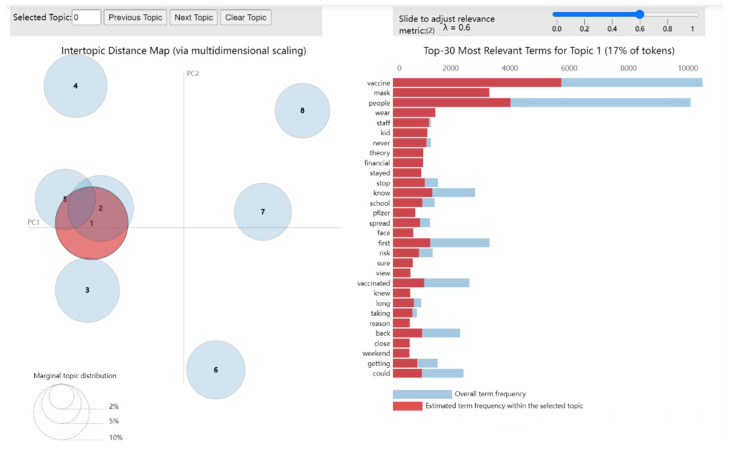
The inter-topic distance map. 1. saliency (term w) = frequency(w)* [sum t p (t|w) * log (p (t|w/p(t))] for topics t; see [68]. 2. relevance (term w|topic t) = λ * p (w|t) + (1 − λ) *p (w|t)/p(w); see [67].

Inter-topic distance maps can help us understand the relationships between topics, and the distance between circles indicates the distribution similarity between topics. Each circle represents a different topic, and the size of the circle means how many documents contain that topic. The number label on the circle represents the order of its size, and the circle with the number 1 is the most critical topic. In Figure 4, eight topics presented the LDA model output distribution of the global view and its most crucial vocabulary associated with the topic. Themes 3, 4, 6, 7, and 8 are relatively independent, while themes 1, 2, and 5 have a significant similarity.

### 4.3. Social Network Analysis of Social Bots and Human Accounts

#### 4.3.1. Social Network Structure

There were 120,118 epidemy-related tweets in this study, and 34,935 Twitter accounts were detected as bot accounts by Botometer, accounting for 29%. In all, 82,688 Twitter accounts were human, accounting for 69%; 2495 accounts had no bot score detected.

In social network analysis, degree centrality is an index to judge the importance of nodes in the network. The nodes in the social network graph represent users, and the edges between nodes represent the connections between users. Based on the network structure graph, we may determine which members of a group are more influential than others. In 1979, American professor Linton C. Freeman published an article titled “Centrality in social networks conceptual clarification“, on Social Networks, formally proposing the concept of degree centrality [69]. Degree centrality denotes the number of times a central node is retweeted by other nodes (or other indicators, only retweeted are involved in this study). Specifically, the higher the degree centrality is, the more influence a node has in its network. The measure of degree centrality includes in-degree and out-degree. Betweenness centrality is an index that describes the importance of a node by the number of shortest paths through it. Nodes with high betweenness centrality are in the “structural hole” position in the network [69]. This kind of account connects the group network lacking communication and can expand the dialogue space of different people. American sociologist Ronald S. Bert put forward the theory of a “structural hole” and said that if there is no direct connection between the other actors connected by an actor in the network, then the actor occupies the “structural hole” position and can obtain social capital through “intermediary opportunities”, thus having more advantages.

Because Gephi is more suitable for processing big dynamic data for observational analysis, it has a powerful visualization function and solid dynamic analysis [70]. Previous studies used Gephi for visualization and network analysis [71,72]. After the processed data is imported into the Gephi software, the algorithm drawing and visualization operations are carried out for the data sets. The information propagation path and law in the Twitter social network composed of social bots and human accounts are further analyzed. This study with users as node data and retweet relationships as edge data; the data is cleaned and processed by python programming imported into Gephi for visualization. Compared with weak connections such as “like” and “follow”, the number of the specific relationship “retweet” may be less than the number of user nodes. However, it has an essential meaning of “diffusion” and can better reflect the willingness of node users. More extensive networks and shorter text tend to increase the number of weak connections, and many node users may not have “edge” connections. After processing, the data contains 100,113 nodes and 86,453 edges.

**Figure 5:**
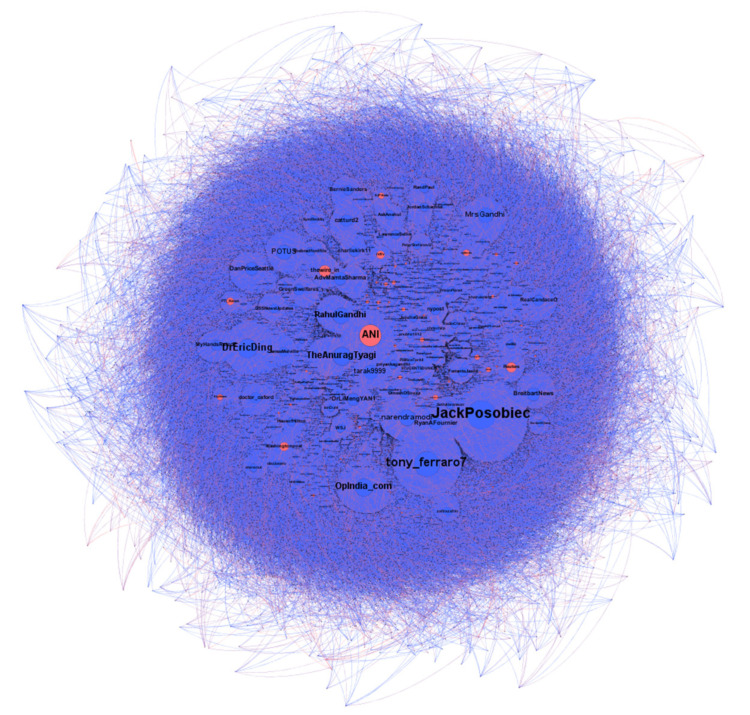
Twitter users’ retweet network.

As is shown in Figure 5, the color of nodes is divided into red and blue to distinguish between social bot accounts and human accounts. According to the bot detection results, accounts with a score over 0.5 are classified as social bot accounts and marked in red. The accounts with a score of less than 0.5 are classified as human accounts and marked in blue. As some public figures and news organizations also have automated manipulation behavior, their scores will be higher than 0.5 after detection. It may be that the operation strategy of the team behind them leads to the concentrated sending time of tweets or the application of Twitter API for automated operation. Therefore, this study also marks such accounts as bot accounts.

**Figure 6:**
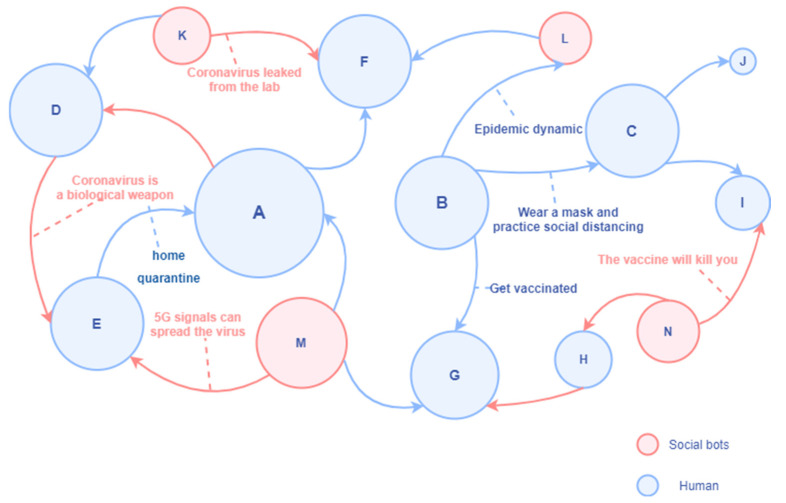
Human-machine communication in the network.

According to the network of discussions on the Twitter platform, this study simplified and simulated it as the information dissemination diagram in the “human-machine” network. As shown in Figure 6, in the social network of “man-machine symbiosis”, the social bots and humans are both senders and receivers of information. There is a mixing and symbiotic relationship between man and machine.

In Figure 6, the node represented by human A is a high-degree centrality account with poor discrimination ability for disinformation and rumors; it is easily affected by misinformation retweeted by social bots. At the same time, it will also refer to the opinions of other persuasive folk opinion leaders in the retweeting process. Human B represents the official institutional account, which has a high in-degree and often pushes the latest news, preventive measures, and suggestions related to COVID-19. Human C represents a human account with high media literacy, which mainly retweets information from information sources with high credibility. It has a solid ability to identify information quality and is not susceptible to the proliferation of social bots. Human D actively creates and spreads rumors and conspiracy theories and only retweets unverified messages that support his views in an attempt to expand the influence. Social bots K, M, and N also spread unverified information (rumors, conspiracy theories, and disinformation) in the communication network without fact-checking. Social bot L may be a social bot of an official agency.

#### 4.3.2. In-Degree Centrality Analysis

Degrees are properties of nodes, but they are related to edges. Without edges, there is no degree. The node’s number of edges is also the node’s degree. In a directed graph, edges have directions, and the degree of a node can be further divided into degree, in-degree, and out-degree. The degree is equal to the sum of the out-degree and in-degree. In this study, the degree of a node is directly related to the retweeting relationship. The higher the in-degree is, the more times it is retweeted by other nodes. The higher the out-degree is, the more it is retweeted.

The top 30 accounts ranking by in-degree are shown in Table 4. Twitter’s verified accounts include media organizations, politicians, industry experts and celebrities. According to the top 30 accounts with the highest click-in degree, they all have a high number of followers. Most of them are official verified accounts, and their original tweets have been retweeted by other users many times, showing the prominent characteristics of opinion leaders.

#### 4.3.3. Out-Degree Centrality Analysis

In this study, the out-degree represents the times of retweeting. The higher the out-degree, the more the node retweets other nodes. According to statistics, the top 30 out-degree Twitter accounts, as shown in Table 5, all nodes are unofficial authentication accounts. Among the most popular accounts, many had the word “bot” in their names, indicating that they were social bots whose identities were public. According to the previous study, social bots can have many good and bad applications. Like any software, they can automate tasks faster than humans, such as automatically publishing news or changing templates for all pages in a category on Wikipedia (for example, Wikipedia-Bot 2015) [73]. Social bots that automate tasks on social platforms are mainly used to spread all kinds of news or publish helpful information, such as weather updates and sports scores, so they must retweet popular tweets frequently.

#### 4.3.4. Betweenness Centrality Analysis

Betweenness centrality measures the degree to which actors control resources. Nodes with higher betweenness centrality play the role of bridge between others. Linton C. Freeman believes that intermediate members of social networks have a more significant interpersonal influence on members at both ends of the path [74]. In other words, information communication in the process of public participation in discussion largely relies on accounts with high betweenness centrality.

Through calculation, discussion of Twitter users’ betweenness centricity ranking data is shown in Table 6. The data in the table indicates that 30 accounts such as “Nitin043”, “Ansaar_Al1”, “Yoursurajnaik”, “SomenMitra3”, and “Sitansh64621089” had high intermediary centrality. It shows that the above users are an essential bridge for spreading epidemy-related information in the corresponding time and play a more prominent role in the “bridging” of control ability and information resources of other nodes, having a specific right to spread. However, the average betweenness centrality value of more than 99% of users is 0; their influence is feeble, and most users are affected by the top accounts to retweet. It indicates that a few node users control the retweeting behavior of many other node users.

Among the accounts that ranked high in betweenness centrality, there were few verified Twitter accounts but more personal accounts. Combined with the core topics in Twitter discussions, it can be speculated that the personalized narratives of ordinary user nodes are more likely to attract other actors to interact in this period. That is, folk opinion leaders have more communication ability in discussing the lab-leak theory. In addition, Botometer detected 30 accounts with high betweenness centrality scores and found that, except for one account (@dev009_sk) that failed to be detected, a total of 19 accounts out of the remaining 29 accounts were suspected to be social bots. It can be seen that social bots played a critical role as a bridge in discussions related to COVID-19.

## 5. Discussion

This study analyzed lab-leak conspiracy theories on Twitter in the context of COVID-19, finding the influence of social bots on social media to manipulate public opinion. According to some findings, while automated accounts are numerous and actively engaged in discussing controversial issues, they generally do not appear to increase human users’ exposure to negative and inflammatory content [47]. However, in this study, we found that social bots played a bridge role in diffusion in the apparent directional topic like “Wuhan Lab”. Previous research also found that social bots play some intermediary roles between elites and everyday users regarding information flow [43]. In addition, verified Twitter accounts continue to be very influential and receive more retweets, whereas social bots retweet more tweets from other users. Studies have found that verified media accounts remain more central to disseminating information during controversial political events [75]. However, occasionally, even the verified accounts—including those of well-known public figures and elected officials—sent misleading tweets. This inspired us to investigate the validity of tweets from verified accounts in subsequent research. It is also essential to rely solely on science and evidence-based conclusions and avoid opinion-based narratives in a time of geopolitical conflict marked by hidden agendas, disinformation, and manipulation [76].

By comparing other studies on conspiracy theories about the origin of COVID-19, we conclude that controlling social bots is an important way to reduce the spread of conspiracy theories. Primarily, two theories about the origin of COVID-19 have circulated widely in China and the West, one blaming the US and the other on the highest-level protection laboratory in Wuhan, the pandemic’s initial epicenter. Both theories claim that biological warfare was attempted. These claims are unsubstantiated by scientific evidence [77]. Previous research has shown that conspiracy theories are reinforced in online communities and that social norms change when conspiracy theories are translated into real-world action. These actions are posted back to social media, where they are reinforced and amplified further, and the cycle continues. Increasing public awareness of conspiracy theories may lead to people viewing them as more popular, potentially normalizing them into the mainstream [16]. At the same time, we should notice the role of social bots in this conspiracy cycle. Secondarily, conspiracy theories can have many negative consequences, including harmful effects on health-related behaviors [78]. As stated in the study, the persistence of COVID-19 conspiracy theories may be one of the reasons for global poor acceptability and ambivalence about COVID-19 vaccines [79]. People’s beliefs about infectious disease conspiracy theories can negatively affect their health behaviors regarding vaccination [80]. Thirdly, selected sources of COVID-19 information and social media connections predicted the origin of COVID-19 beliefs [81].

Therefore, the government, the medical community, social media platforms, and media organizations must work together extensively to counteract the spread of conspiracy theories and their detrimental effects. To be specific, flagging bot accounts and the misinformation they share could be an effective strategy to prevent the spread of false information online [41]. Platforms can inform users before they encounter false or misleading information, or they can “correct” information to disprove conspiracies [82]. To effectively communicate with the public, health authorities and the media should adopt a more critical rhetorical strategy [83].

At the present time, the social bot is embedded in the human communication network, which makes the information communication in the social platform show the pattern of human-machine symbiosis. At the same time, social bots have become an essential driving force for the diffusion of low-credibility content, affecting the information acquisition and dissemination of regular human accounts. Through the interconnection and centralized retweeting of many topics, social bots can complete the public opinion manipulation process of setting topics, strengthening frameworks, network cooperation, and influencing audiences quickly. In the multiple rotations of social bots, conspiracy theories and disinformation mixed with other network information are spread more widely, causing more ordinary users to retweet, thus strengthening the manipulation of public opinion. Under the joint action of topic manipulation and technology manipulation, when ordinary users enter the “human-machine” network controlled by hidden forces, they must accept the influence and domination of the controlled information. Therefore, although the social bot individual is “small”, it has become a “super spreader” with strategic significance. As an intelligent communication subject in the social platform, it conspired with the discourse framework in the mainstream media to form a hybrid strategy of public opinion manipulation.

## 6. Conclusions

This study visually analyzed the human-machine interaction misinformation propagation process and revealed the role of social bots, providing practical guidance for health authorities on how to combat rumors and conspiracy theories in the context of the pandemic in the future.

Social bots, which account for 29% of all accounts, were observed to have participated in Twitter discussions during the peak of the Wuhan lab-leak theory. We discovered that while verified accounts have a greater direct influence on the majority of users in retweet networks, social bots have a greater indirect influence than humans. They do not set the agenda, but they do much to distribute it. Additionally, a small number of nodes, or headers, control the majority of users’ retweets. Unverified accounts played a greater role than any other type of intermediary in the dissemination of conspiracy-related topics. As a result, controlling the actions of social bots and spreading misinformation at their source is critical to reducing their impact.

### Limitations and Future Directions

Twitter data are appropriate for a variety of research issues and have been used in published studies on a number of significant subjects [84,85,86,87]. However, using Twitter as a single data source has advantages and disadvantages. On the one hand, based on this paper’s research purpose and tool limitations, we chose the Twitter platform, which is more friendly to researchers and has mature bot detection tools, for research. On the other hand, the social media used by people in daily life are very diverse, including some video platforms. How information flows and functions among these platforms is a new problem worth studying, which is also the limitation of this research. Moreover, little is known about the sampling quality of the Twitter API and third-party platforms, and there is little opportunity to verify how the back-end samples, so there is some data bias [88].

In addition, how individual users on social platforms perceive and are affected by the behavior of social bots needs to be further studied. Whether the influence of social bots is mainly focused on individual users or the overall network structure remains to be explored, related to the measurement of social impact and network influence of social bots. At the same time, this research can further innovate from data, methods, and case selection, and expand the study by combining political theories and information ethics.

## Figures and Tables

**Table 1:** Top 20 high-frequency keywords in tweets.

No.	Keyword	Frequency	No.	Keyword	Frequency
1	people	9039	11	home	3284
2	vaccine	8806	12	mask	3208
3	lockdown	6509	13	first	3104
4	case	4965	14	China	3020
5	India	4606	15	state	2864
6	need	3909	16	please	2797
7	death	3903	17	know	2792
8	Wuhan	3903	18	vaccination	2729
9	government	3876	19	life	2608
10	health	3436	20	country	2577

**Table 2:** Text preprocessing before and after contrast sample.

Before Text Preprocessing	After Text Preprocessing
RT @voguemagazine: Read how the healing power of Lodge 49 helped one writer break through their pandemic fog. https://t.co/V1JOVGKTXy accessed on 9 October 2022	[vogue magazine, read, healing, power, lodge, helped, writer, break]

**Table 3:** Eight topics of LDA model.

No.	Topic	High-Frequency Words
0	coronavirus origin-tracing	virus, china, wuhan, since, beginning, chinese, fauci, origin, trump, biden
1	Prevention and vaccine	lockdown, vaccine, best, headline, myhandsratede, since, second, government, rate, johnson
2	Social influence	home, stay, care, vaccine, people, lockdown, need, food, worker, doctor
3	Family influence	child, lost, parent, student, narendramodi, please, exam, govt, future, government
4	Virus variation	case, death, health, india, lockdown, total, variant, vote, indian, died
5	Preventive measures	vaccine, people, mask, wear, know, first, staff, kid, never, stop
6	Health effects	family, every, patient, panic, positive, friend, hospital, someone, buying, president
7	The public interest	people, lockdown, thank, first, country, free, vaccinated, around, social, mean

**Table 4:** Top 30 twitter accounts by in-degree.

No.	Account	In-Degree	No.	Account	In-Degree
1	JackPosobiec *	504	16	RyanAFournier *	213
2	tony_ferraro7	442	17	DrLiMengYAN1	208
3	ANI *	377	18	catturd2	206
4	BIGHIT_MUSIC *	344	19	thewire_in *	200
5	DrEricDing *	335	20	doctor_oxford *	195
6	TheAnuragTyagi	314	21	GreenSwelfares	187
7	OpIndia_com *	310	22	RealCandaceO *	183
8	RahulGandhi *	280	23	charliekirk11	176
9	narendramodi *	276	24	BernieSanders *	175
10	MrsGandhi *	262	25	nypost *	175
11	POTUS *	255	26	MyHandsRatedE	175
12	tarak9999 *	247	27	Reuters *	171
13	DanPriceSeattle *	226	28	DSSNewsUpdates *	167
14	BreitbartNews *	224	29	JamesMelville *	166
15	AdvMamtaSharma	223	30	AskAnshul *	163

* Twitter’s verified accounts.

**Table 5:** Top 30 Twitter accounts by out-degree.

No.	Account	Out-Degree	No.	Account	Out-Degree
1	CoronaUpdateBot	21	16	CyberSecurityN8	9
2	fengmanlou11	19	17	DipMond81427857	9
3	viralvideovlogs	18	18	Ken34205423	9
4	HKLongman	16	19	PhotoLawn	9
5	Covid19Help10	15	20	TALI189	9
6	world_news_eng	14	21	peterandann	9
7	BotJammu	13	22	trackntracer	9
8	SLRTBot	13	23	roadtoserfdom3	8
9	MiniMooJack	12	24	PankajC47069041	8
10	aOraxoSizcr8Dlh	12	25	AyanAdhikari13	8
11	KRS_Deshsevak	11	26	CoronaBot20	8
12	scouts_uk	11	27	KRISHANMOHANKR6	8
13	IRFANNKPCC	10	28	MKSafdar	8
14	hekhwthktiingh1	10	29	MonaSmitte	8
15	B0tSci	9	30	Rubydawne1	8

**Table 6:** Top 30 Twitter accounts by betweenness centrality.

No.	Account	Betweenness Centrality	No.	Account	Betweenness Centrality
1	Nitin043	440.5	16	dev009_sk	123
2	Ansaar_Al1	408	17	chrischirp	111
3	yoursurajnaik	363	18	souravramyani	111
4	SomenMitra3	355.5	19	RavinderKapur2	104
5	Sitansh64621089	349.5	20	sonumehrauk	104
6	PankajC47069041	322.5	21	gourav_chakr	99
7	Ndlotus1	279.5	22	doctor_oxford*	94
8	SPanda4485	269	23	RajVB6	93
9	imChikku_	256	24	fascinatorfun	89
10	Magamiilyas	206.5	25	chinmoyee5	83
11	Thecongressian	197.5	26	fekubawa	81
12	RamshettyVishnu	186	27	ukiswitheu	80
13	Drmandakini3	136	28	BalharaAbhijeet	78
14	DrINCsupporter	126	29	erdocAA	75
15	Jagjit_INC	125	30	Abhishe32226771	75

## Data Availability

Not applicable.
